# Application of Portsmouth modification of physiological and operative severity scoring system for enumeration of morbidity and mortality (P-POSSUM) in pancreatic surgery

**DOI:** 10.1186/1477-7819-6-39

**Published:** 2008-04-09

**Authors:** Appou Tamijmarane, Chandra S Bhati, Darius F Mirza, Simon R Bramhall, David A Mayer, Stephen J Wigmore, John AC Buckels

**Affiliations:** 1 Queen Elizabeth hospital, liver unit, Birmingham, UK

## Abstract

**Background:**

Pancreatoduodenectomy (PD) is associated with high incidence of morbidity and mortality. We have applied P-POSSUM in predicting the incidence of outcome after PD to identify those who are at the highest risk of developing complications.

**Method:**

A prospective database of 241 consecutive patients who had PD from January 2002 to September 2005 was retrospectively updated and analysed. P-POSSUM score was calculated for each patient and correlated with observed morbidity and mortality.

**Results:**

30 days mortality was 7.8% and morbidity was 44.8%. Mean physiological score was 16.07 ± 3.30. Mean operative score was 13.67 ± 3.42. Mean operative score rose to 20.28 ± 2.52 for the complex major operation (p < 0.001) with 2 fold increase in morbidity and 3.5 fold increase in mortality. For groups of patients with a physiological score of (less than or equal to) 18, the O:P (observed to Predicted) morbidity ratio was 1.3–1.4 and, with a physiological score of >18, the O:P ratio was nearer to 1. Physiological score and white cell count were significant in a multivariate model.

**Conclusion:**

P-POSSUM underestimated the mortality rate. While P-POSSUM analysis gave a truer prediction of morbidity, underestimation of morbidity and potential for systematic inaccuracy in prediction of complications at lower risk levels is a significant issue for pancreatic surgery

## Background

Pancreato-duodenectomy (PD) is associated with high incidence of morbidity and mortality. Mortality rates vary widely from 0% to 28% [1-4], with specialist centres performing high volume surgeries reporting comparatively lower complications and deaths[3]. However, the incidence of morbidity after PD is still high, even in specialist centres[2,3,5]. For complex operations, the most common outcome measured is mortality. To meaningfully interpret the outcome measurement the incidence of complications following complex operations must be analysed. Crude rates of morbidity and mortality do not justify these measurements, do not reflect the standards of care and technical expertise required for the perioperative needs of complex cases such as those in the hepato-biliary and pancreatic surgery and may be misleading because such rates make no allowance for differences in case mix and fitness of patients[6]. Various scoring systems such as the ASA (American Society of Anaesthesiologists) score, APACHE 2 (Acute Physiology and Chronic Health Evaluation), POSSUM (Physiological and Operative Severity Scoring System for Enumeration of Morbidity and Mortality) and its Portsmouth modification (P-POSSUM) are in place to assess the risks involved for patients in various specialities.

In contrast to APACHE 2, POSSUM and its modifications take operative findings into consideration [7]. Since it's first report in 1991[8], POSSUM and its modifications have been recognised as highly effective for surgical audit purposes. It is calculated based on 12 physiological and 6 operative parameters derived originally from the multivariate analysis of 48 physiological and 14 operative variables, and has a 4-level exponential score of severity. Since the POSSUM score has been noted to over-predict mortality especially with minor procedures, the Portsmouth POSSUM (P-POSSUM) model was developed which utilises a linear method of analysis providing a 'good fitness' on the observed mortality [9].

Studies to evaluate the POSSUM and P-POSSUM scores in hepato-biliary and pancreatic surgery[10] were hampered by[11] small numbers of patients and the widely varied case mix meaning that the overall interpretation becomes difficult especially when it is applied to PD which is associated with significant morbidity and mortality in comparison to other hepato-biliary surgeries.

To fully evaluate the impact of P-POSSUM analysis on the post operative morbidity and mortality rates this study incorporates a large consecutive group of patients who had PD in a tertiary referral centre.

## Patients and methods

Prospective data of 241 consecutive patients who underwent PD with or without pylorus preservation between January 2002 and September 2005 at the Liver Unit, Queen Elizabeth Hospital, Birmingham were retrospectively updated and analysed. All patients who initially were listed for PD but had total pancreatectomy for atrophic pancreas, multi-focal disease or positive resection margin on frozen section were excluded.

The physiological and operative score was calculated for each patient using P-POSSUM analysis via an online risk score calculation program [12]. For the operative score, PD with or without pylorus preservation was assigned as *'major' *and where venous resection and/or resection of adjacent viscera occurred the *'complex major' *category was assigned. Other operative parameters include number of procedures, total blood loss, peritoneal soiling, malignant status and timing of surgery. Physiological score was calculated using the parameters including age, cardiac signs, respiratory signs, systolic blood pressure, pulse rate, Glasgow coma scale, serum urea, serum sodium, serum potassium, haemoglobin, white cell count and electrocardiogram. Post-operative morbidity was subdivided into minor (delaying discharge), intermediate (requiring non-invasive intervention, such as starting on antibiotics, anticoagulation for atrial fibrillation etc) and major (life-threatening complications or requiring invasive intervention such as endoscopic, interventional radiological or surgical intervention)[11]. 30 day postoperative mortality was recorded. For the purpose of logistic regression analysis, severe morbidity and death were combined to form a dichotomous variable. We have used the term 'severe morbidity' instead of 'major morbidity' to avoid confusion with another variable used in the logistic regression analysis (the extent of pancreatic surgery – major or complex major).

Statistics: Mann-Whitney test for non-parametric data and Kendall tau-b test statistic for the ordinal data were used. Any variable whose univariate test had a *P*-value of <0.25 was considered for the multivariate analysis. Step-wise logistic regression analysis was performed to identify the multivariate model for the dependent variable *'severe complication and death'*. Statistical Package for the Social Sciences" version 12 for Windows (SPSS, Chicago, IL, USA) was used for the above analysis.

## Results

**Figure 1:**
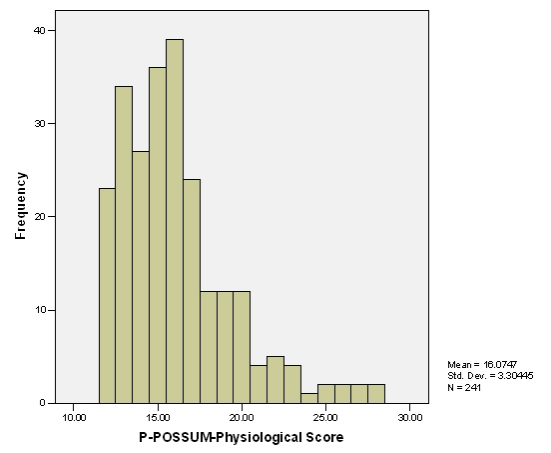
Distribution of patients according to Physiological Score.

**Figure 2:**
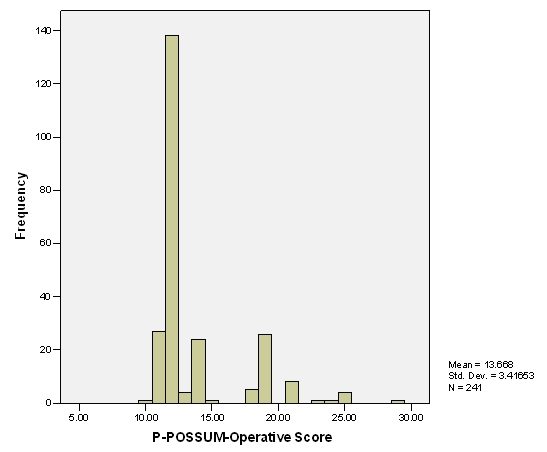
Distribution of patients according to Operative Score.

Demographic characteristics are shown in Table 1. There was no significant difference in the median physiological scores and operative scores between different aetiological groups (Table 2). From among 205 patients who underwent pancreatoduodenectomy with or without pylorus preservation, 13 (6.3%) died within 30 days of surgery. Four patients (15.4%) died out of 26 patients who had superior mesenteric vein resection. Eight patients had adjacent viscera resection due to local tumour involvement and two of them died during their admission. Two patients who had venous resection as well as adjacent viscera resection survived. The distribution of patients according to physiological and operative scores is shown in fig 1 &2 respectively. Nearly 50% of major complications were of gastrointestinal origin (Table 3). The overall observed 30 days postoperative mortality was 7.8% and morbidity was 44.8%.

**Table 1:** Patient characteristics

**Patient characteristics**	**Number**
Median Age (range)	64.06 (21.7, 84.5)
Male: Female	135:106
Median Hospital stay (range)	10 (3, 73)
Median ITU stay (range)	0 (0, 31)
30 days Mortality (%)	19 (7.8%)
Minor/intermediate morbidity (%)	56 (23.2%)
Major morbidity (%)	52 (21.6%)

ITU, Intensive Therapeutic Unit

**Table 2:** Aetiology and P-POSSUM Scores

**Diagnosis**	**Physiological Score**		**Operative Score**	
	
	**Mean ± SD**	**Median**	**Mean ± SD**	**Median**
Adenocarcinoma HOP	16.16 ± 3.31	16.00	13.76 ± 3.15	12.00
Ampullary carcinoma	15.80 ± 3.76	15.00	12.74 ± 2.06	12.00
Carcinoma of lower CBD	16.52 ± 2.76	16.00	14.86 ± 4.39	12.00
Duodenal carcinoma	16.38 ± 2.50	16.00	13.76 ± 4.22	12.00
Others-Malignant	15.87 ± 3.18	15.00	15.37 ± 4.74	12.00
Others-Benign	15.88 ± 3.40	15.00	12.36 ± 2.91	11.00

P-POSSUM, Portsmouth Modification of Physiological and Operative Severity Score for the enumeration of Mortality and morbidity; HOP, Head of pancreas; CBD, Common Bile Duct; SD, Standard Deviation

**Table 3:** Summary of Morbidity

**System**	**Minor/Intermediate**	**Major**
Respiratory	6	11
Cardiac	16	3
Gastrointestinal	13	36
Renal	4	4
Septicaemia	0	8
MSOF	0	8
Wound	12	0
Others	9	1

Note: A given patient may have complications of different magnitude in one or more systems. MSOF, Multiple System Organ Failure

The overall mean physiological score was 16.07 ± 3.30. The overall mean operative score was 13.67 ± 3.42. However, the mean operative score rose to 20.28 ± 2.52 for the complex major operation (p < 0.001) with 2 fold increase in morbidity and 3.5 fold increase in mortality, in comparison to those who underwent PD without any venous or additional visceral resection.

The observed to predicted ratio (O:P) in terms of overall morbidity was 1:4 for groups with physiological score ≤ 15.00. However this ratio seems to be closer to 1 as the physiological score increases to above 18.00. The average O:P ratio for the postoperative mortality was 3:4. In effect, P-POSSUM under predicted mortality (Table 4). The observed morbidity was significantly greater than the predicted morbidity (p < 0.001) and the observed mortality was significantly greater than the predicted mortality (p < 0.001), when Hosmer-Lemeshaw goodness-of-fit test was applied. Hence the P-POSSUM risk morbidity and mortality scores were not good predictors of outcome at least in our data.

**Table 4:** Stratification of morbidity and mortality according to P-POSSUM Physiological score

**Physiological Score**	**Morbidity (Predicted)**	**Morbidity (Observed)**	**O:P ratio**	**Mortality (Predicted)**	**Mortality (Observed)**	**O:P ratio**
< = 15.00	24.92	35.8	1.4	1.08	(5.8)	5.4
15.1 – 18.00	35.18	46.7	1.3	1.90	(6.7)	3.5
18.1 – 21.00	47.23	50.0	1.1	3.15	(14.3)	4.5
21.1 – 24.00	56.70	70.0	1.2	6.82	(10.0)	1.5
24.00+	73.85	87.5	1.2	12.12	(25.0)	2.1

O:P, Observed to Predicted Ratio.

Factors predicting severe complications and death include haemoglobin (Hb) (p = 0.013), white cell count (WCC) (p = 0.059), albumin (p = 0.04), the extent of pancreatic surgery (complex major when venous or additional organ resections were performed) (p = 0.033) and P-POSSUM physiological score (p = 0.001) as identified by univariate analysis whereas logistic regression analysis revealed that only P-POSSUM physiological score (p = 0.005 with Exp(B) = 1.138, 95% CI for Exp(B) = 1.040–1.245) and WCC (p = 0.010 with Exp(B) = 1.150, 95% CI for Exp(B) = 1.034–1.280) were significant predictors whereas the remaining variables identified by the univariate analysis were not significant in the logistic regression model (Hb – 0.539, albumin – 0.132 and the extent of pancreatic surgery – 0.661). Combined variable of physiological score and bilirubin level (grouped into those above or below 300 μmol) did not show any significant effect (p = 0.506) in this regression model.

## Discussion

Surgical audit is important both as an educational process and as a means of assessing the quality of surgical care. Since the specialist operations such as PD are associated with high incidence of morbidity and significant risk of mortality, the authors felt that there was a need to perform the risk stratification in order to assess our postoperative outcome results with P-POSSUM score which has already been well validated in other specialities. Kocher *et al *reported the highest risk of operative morbidity for PD after having adjusted for the type of other confounding variables (O:P 2.27, 95%CI: 1.07–9.97) in comparison with the right hepatectomy, which was treated as the reference category[11] in their series. While operative mortality has decreased in specialist centres, morbidity remains high for pancreatic surgery[3,5] and perhaps represents a more objective parameter of quality of care[10].

Any comparative scoring system might make poor results look better by over predicting morbidity and mortality. Various scoring systems were evaluated in different specialities of general surgery to standardize patient related parameters and compare performance in a risk-adjusted manner[7,10]. The POSSUM and its modifications have been applied to various sub-specialities of general surgery including vascular, colorectal and thoracic surgery[6,13-15].

The original POSSUM scoring system as devised by Copeland et al[8] has been criticized because of it's tendency to over predict the morbidity and mortality and this has been attributed to the exponential method of analysis and it is difficult to give a risk score to an individual patient by this system[16]. On the contrary, P-POSSUM uses the linear method of analysis, which is a standard method described by Hosmer and Lemeshow[17] and the risk assessment applies to an individual patient and is simpler to use[18].

The lowest possible POSSUM physiological and operative scores are 12 and 6 respectively, with which the predictor equation gives a mortality value of 1.1%[19] and for P-POSSUM a value of 0.2%[21,20]. Analysing uncomplicated surgeries using P-POSSUM resulted in over prediction of morbidity and mortality rates[19,20] whereas analysis of patients who underwent PD in this series shows under prediction of those outcomes (Table 4).

The mortality rate was 6.4% for 219 (91%) of patients with a mean physiological score of 15 in our series and for the remaining 22 (9%) patients with a physiological score of 21 or above, it was 22.7%, a more than three fold increase in the mortality rate. While our morbidity and mortality figures remained well within the range published in the literature, the observed rates were much higher than the predicted results. These findings may well be the result of assigning different levels of importance to the parameters required to calculate the operative and physiological scores. On the other hand, these results may actually mean that the patients with high scores should be carefully evaluated before subjecting them to major surgical intervention.

For groups of patients with a physiological score of ≤ 18, the O:P (observed to Predicted) morbidity ratio was 1.3–1.4 and, for those with a physiological score of >18, the O:P ratio was nearer to 1 in terms of overall complications (Table 4). In effect, while P-POSSUM analysis gave a truer prediction of morbidity than mortality in our series of patients, underestimation of morbidity and potential for systematic inaccuracy in prediction of complications at lower risk levels is a significant issue for pancreatic surgery. The operative score for PD was achieved through the following criteria: *major operation *for operative severity, *1(one) *for number of procedures, *minor *for peritoneal soiling, *positive *or *negative *for lymph nodal metastases, *elective *for mode of surgery and blood loss *as appropriate*. The average operative score in our group was 13.67 ± 3.4 (median 12, minimum 10 and maximum 29). Whereas the mean operative score in Copeland's original study of general surgical patients was only 6[8]. Interestingly, Khan AW *et al*[10] had a much higher operative score (median 22) in their group of 50 patients undergoing PD which resulted in higher predicted morbidity and mortality values. It is possible to obtain this level of operative score in our group of patients by merely assigning *'complex major' *rather than *'major' *for the operative severity, which resulted in the higher incidence of predicted morbidity and mortality. We have used the *complex major *category for the subgroup of patients who required PD with superior mesenteric/portal vein resection and/or adjacent viscera resection with a resultant mean operative score of 20.28 ± 2.52 (3.5 fold increase in the rate of mortality and 2 fold increase in morbidity). On the other hand, for those who had their operation without any additional venous or visceral resection, the operative score was 12.80 ± 2.42. Hence our observed rates of complications and death rates seemed to be higher compared to the predicted morbidity and mortality rates in comparison to that quoted by Khan AW et al[10]. The presence of malignancy and nodal metastasis may not be a useful discriminant for calculating operative score as their effect is only minimal [5].

**Figure 3:**
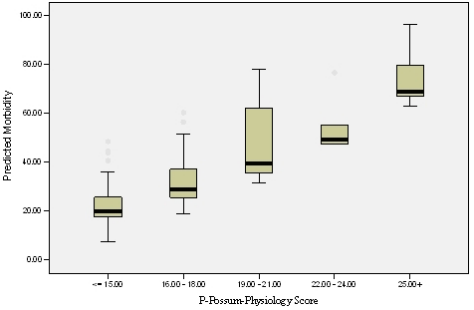
Stratification of morbidity according to physiology score Horizontal lines within boxes, boxes and error bars represent median, interquartile range and range respectively. P < 0.001 (Kruskal Wallis Test).

While the operative score has an element of subjective assessment, the physiological score can be calculated with easily available parameters with very little subjective bias. However, confusion may arise in the interpretation of electrocardiogram (ECG) criteria [16]. Despite the pitfalls mentioned, the physiological score alone may be used as a tool to quantify the risk of morbidity (Figure 3) and mortality while obtaining informed consent. Logistic regression analysis confirmed that the physiological score was the most important factor (p = 0.005) in the equation with *major complication and death *as a dependent variable. Interestingly, the overall operative score did not have any significance in the multivariate model although the extent of pancreatic surgery was one of the significant univariate factors identified. This is probably due to the fact that the mean operative score for the group needing (36 patients) venous and or additional organ resections was 20.28 ± 2.52 with 3.5 fold increase in the rate of mortality and 2 fold increase in morbidity compared to the mean operative score of 12.80 ± 2.42 for the group without such additional resections. In addition to this, WCC had also been shown to have significant impact (p = 0.01) on the outcome, although WCC itself is one of the parameters used for calculation of the physiological score.

## Conclusion

There were limitations to this study because of the retrospective update and analysis of the prospectively collected data. Although the findings were from a single centre with a large hospital volume, these results need to be validated by a similar analysis from another centre. Results of statistical analysis have never intended to affect the decision to operate; this decision must be based on clinical expertise. Due to the need to standardize data collection and stratify the risks involved in operations such as PD, scoring systems such as P-POSSUM should be used prospectively. To avoid the pitfalls in calculating these scores, there needs to be a standard protocol to decide categorisation of operations as major or complex major as this alone can dramatically influence the operative score and predicted outcomes. Only through universal standardisation of criteria can meaningful comparison between regional centres be achieved. It must also be remembered that the P-POSSUM scoring whilst predicting 30 day outcomes does not provide any indication of the prognosis.

## Competing interests

The author(s) declare that they have no competing interests.

## Authors' contributions

AT – Designed the study and prepared the manuscript, statistic calculations; CSB Collection of data and preparation of data bank and preparation of manuscript. DFM, SRB, and DAM Concept and design, supervision; SJW – manuscript correction and supervision, JACB – Concept and design and correction of manuscript,
